# Artificial intelligence on COVID-19 pneumonia detection using chest xray images

**DOI:** 10.1371/journal.pone.0257884

**Published:** 2021-10-14

**Authors:** Lei Rigi Baltazar, Mojhune Gabriel Manzanillo, Joverlyn Gaudillo, Ethel Dominique Viray, Mario Domingo, Beatrice Tiangco, Jason Albia

**Affiliations:** 1 Data-Driven Research Laboratory (DARE Lab), Institute of Mathematical Sciences and Physics, University of the Philippines Los Baños, Los Baños, Philippines; 2 Domingo Artificial Intelligence Research Center (DARC Labs), Pasig City, Philippines; 3 Computational Interdisciplinary Research Laboratories (CINTERLabs), University of the Philippines Los Baños, Los Baños, Philippines; 4 Department of Medicine, The Medical City, Pasig City, Philippines; 5 National Institute of Health, College of Medicine, University of the Philippines, Manila, Philippines; 6 Division of Medicine, The Medical City, Pasig City, Philippines; Newcastle University, UNITED KINGDOM

## Abstract

Recent studies show the potential of artificial intelligence (AI) as a screening tool to detect COVID-19 pneumonia based on chest x-ray (CXR) images. However, issues on the datasets and study designs from medical and technical perspectives, as well as questions on the vulnerability and robustness of AI algorithms have emerged. In this study, we address these issues with a more realistic development of AI-driven COVID-19 pneumonia detection models by generating our own data through a retrospective clinical study to augment the dataset aggregated from external sources. We optimized five deep learning architectures, implemented development strategies by manipulating data distribution to quantitatively compare study designs, and introduced several detection scenarios to evaluate the robustness and diagnostic performance of the models. At the current level of data availability, the performance of the detection model depends on the hyperparameter tuning and has less dependency on the quantity of data. InceptionV3 attained the highest performance in distinguishing pneumonia from normal CXR in two-class detection scenario with sensitivity (Sn), specificity (Sp), and positive predictive value (PPV) of 96%. The models attained higher general performance of 91-96% Sn, 94-98% Sp, and 90-96% PPV in three-class compared to four-class detection scenario. InceptionV3 has the highest general performance with accuracy, F1-score, and g-mean of 96% in the three-class detection scenario. For COVID-19 pneumonia detection, InceptionV3 attained the highest performance with 86% Sn, 99% Sp, and 91% PPV with an AUC of 0.99 in distinguishing pneumonia from normal CXR. Its capability of differentiating COVID-19 pneumonia from normal and non-COVID-19 pneumonia attained 0.98 AUC and a micro-average of 0.99 for other classes.

## Introduction

A supplementary to reverse transcription polymerase chain reaction (RT-PCR) in screening COVID-19 is imperative to augment the current global strategies in mitigating its continuous spread and potential future outbreak. Although RT-PCR testing is precise and considered as the gold standard for COVID-19 diagnosis, it is not easily accessible and scalable because of the costs and operational requirements [1–3]. Due to this limitation, radiologic-based approaches have been widely adopted for the initial screening of suspected cases. Preliminary studies showed that analysis of chest x-ray (CXR) images might lead to better sensitivity and specificity than RT-PCR-based diagnosis. Furthermore, the misdiagnosis rate of COVID-19 is very high and the misdiagnosis cost is expensive [4]. While the wide availability of CXR machines make it an attractive option for rapid and extensive screening, many radiologists had difficulty reading CXR due to the indistinct manifestation of radiological features such as consolidation and hazy increased opacities [5–8]. A technology-driven solution is to develop an artificial intelligence (AI)-based detection system that will facilitate an automated, accurate, and rapid COVID-19 pneumonia screening based on CXR images.

In recent years, medical diagnosis using AI-driven systems have demonstrated remarkable progress in assisting radiologists and clinicians for disease detection, characterization, and monitoring. The automated nature of AI to recognize intricate patterns in radiologic images and its ability to provide quantitative assessment offer an efficient and scalable mechanism to augment the current diagnostic workflow in the hospitals and ambulatory testing centers. There were preliminary works that utilized AI-driven methodologies to assist radiographic examinations in identifying the visual indicators highly associated with COVID-19. Wang et al. [2] introduced COVID-Net, a convolutional neural network (CNN) designed to detect COVID-19 cases using CXR images. The COVID-Net was trained using 13,800 CXR images to identify COVID-19-related cases and attained 92.6% accuracy and sensitivity of 97.0% (normal), 90.0% (non-COVID-19 pneumonia) and 87.1% (COVID-19). Recently, Basu et al. [9] trained AlexNet, VGGNet, and ResNet on a dataset consisting of 108, 379 CXR images derived from the US National Institute of Health to classify between diseased and normal CXR. These models were subsequently retrained via transfer learning using 1, 277 images and achieved 90.13% accuracy in distinguishing normal, other diseases, pneumonia, and COVID-19. A non-conventional approach in using transfer learning is to utilize the pretrained architectures as feature extractors. Turkoglu [10] extracted features using AlexNet, selected features using Relief, and classified the images using support vector machines (SVM) whereas Montalbo [11] concatenated the extracted features from two truncated Densenets and added a classification head. Another study trained AlexNet, GoogleNet, and ResNet and made the final prediction via majority voting [12]. In addition, several studies [13–16] have shown successful model development via transfer learning by incorporating data augmentation strategies such as rotation, translation, flipping and scaling to increase the number of training instances. Moreover, several works, albeit adopting different base architectures and development strategies, have also illustrated the potential of AI in detecting COVID-19 pneumonia using CXR images [4, 5, 9, 13, 14, 17–21].

While the studies mentioned above have shown high classification performance of AI models, several issues have emerged concerning its clinical applicability. The most apparent issue is the data quality and quantity. The majority of the datasets used in developing AI models were derived from public repositories. These datasets were aggregated from various sources and typically do not include metadata and associated clinical information that may allow researchers to verify its validity. Moreover, the absence of demographic characteristics and other potential risk factors impedes an alternative approach in examining these medical images. Considering the recency of the pandemic, the number of positive cases has also been limited, resulting in models trained on a highly imbalanced dataset. Another issue is the lack of information on how these datasets were generated, thereby restricting researchers to design a suitable retrospective study to evaluate the dependence of the results on the population size, gender, age groups, and race, among others.

In this work, we address some of the issues by generating our own dataset through a well-designed retrospective clinical study to augment the dataset available in public repositories. We pursued a comprehensive model development workflow by manipulating dataset distribution and introducing different detection scenarios to look for an easily deployable model for practical use. More precisely, the contributions of this article are as follows:

- From a clinical standpoint, the AI models could be used as a tool to assist radiologists screen suspected COVID-19 patients, thereby shortening the waiting time for clinical decisions—whether RT-PCR is necessary for a confirmatory step or to remove these patients from the suspected lists. We note that the development of AI-driven detection of COVID-19 pneumonia does not intend to replace the RT-PCR test as it is the gold standard in diagnosing COVID-19. Rather, AI-driven detection aims to augment the inaccessibility of RT-PCR machines in many countries.
- From a methodical perspective, this study illustrates the potential of an AI-driven system for pneumonia (COVID-19, viral, and bacterial) detection considering a more realistic data distribution. We provide different level of detection scenarios which could be adopted as a development approach for a more localized clinical deployment. We used a wide variety of metrics, e.g., accuracy, sensitivity, specificity, negative predictive value, positive predictive value, negative and positive likelihood ratio, confusion matrix, and area under the receiver operating characteristic curve to rigorously evaluate the general as well as the per-class performance of models. Furthermore, visual explanations of the prediction were generated using gradient-based class activation maps (Grad-CAM) to facilitate analysis of the region of interest.
- From a data perspective, this study provides a clinically validated dataset to augment the existing publicly available datasets such as [3, 22], among others, that were used by the research community to develop AI-driven pneumonia detection models. To our knowledge, during the course of this study, we utilized the highest number of COVID-19 positive cases in developing the detection models, thereby minimizing issues on the class imbalance dataset.

## Model development

**Fig 1:**
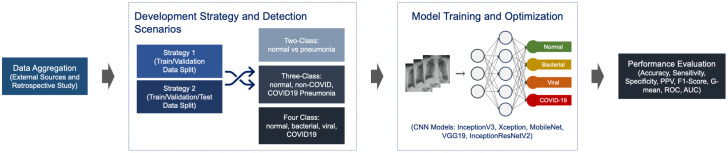
Model development workflow.

The overall development framework adopted in this study is shown in Fig 1. In this section, we describe: (i) study settings and data aggregation from our retrospective clinical study and external sources, (ii) development strategy and detection scenarios introduced to develop the models, (iii) model training via transfer learning and base architecture selection, and (iv) hyperparameter tuning and performance evaluation.

### Study setting and data aggregation

Upon the approval of The Medical City Institutional Review Board (TMC IRB), a database of patients with a CXR done from April 11 to June 1, 2020, as well as a COVID-19 RT-PCR swab done within three days of the CXR, was generated by the TMC IT team. This database contains the patients’ hospital ID number, age, sex, and an assigned study participant ID number. The hospital ID numbers were used by the study clinicians to access patients’ medical records and obtain the clinical information needed through a retrospective chart review. The TMC IRB waived the requirement for informed consent before accessing the medical records. The study clinicians had no direct contact with the patients during the course of the study, and no patient names were encoded or used in data analysis. In addition, the patients’ names were removed from the CXR images metadata.

This internally generated dataset consists of 1,171 CXR images from a total of 821 cohorts with the following inclusion criteria: (i) the age of the participants must be 18 years and above, (ii) have RT-PCR results and CXR images, (iii) were admitted to TMC under the care of infectious disease and/or pulmonary specialists, and (iv) the demographic and clinical data are available, including clinical diagnosis or indicators in the CXR images. 430 (52%) and 391 (48%) of the cohorts are male and female, respectively. The age of the cohorts ranges from 23–100 years old, of which 63% of the patients with COVID-19 pneumonia are older than 60 years old. Out of the total instances, 335 are normal, 194 are abnormal non-pneumonia, 565 are non-COVID-19 pneumonia, and 77 are COVID-19 pneumonia. We note that while 135 instances had positive RT-PCR results, only 77 are labelled COVID-19 pneumonia, and the rest were either normal or abnormal non-pneumonia. We aggregated CXR dataset designed for pneumonia detection studies from several external sources to augment our internal dataset. Table 1 shows the summary of the dataset and their corresponding labels used in this study to develop the COVID-19 pneumonia detection models. Tables 1 and 2 in S1 Appendix show summary statistics of the clinical information of the cohorts and the description and sources of the externally aggregated dataset, respectively. The dataset used in this study is organized and can be accessed through https://github.com/lpbaltazar/COVID-CXR-AI.

**Table 1:** Summary of the dataset used to develop the detection models.

Class Label	Internal	External	Total
Normal	335	3,258	3,593
COVID-19 Pneumonia	77	552	629
Viral Pneumonia	-	1,505	1,505
Bacterial Pneumonia	-	2,786	2,786
Abnormal Non-Pneumonia	194	-	194
Non-COVID-19 Pneumonia	565	-	565
**Total**	**1,171**	**8,101**	**9,272**

### Development strategy and detection scenarios

We adopted two development strategies to evaluate the performance of the models on different dataset distribution. Similar to the earlier works [13, 17–19], the first strategy involves the maximization of the data to build the model, i.e., the dataset was divided into 80% training and 20% validation. This strategy exposes the base architecture to more data during optimization thereby allowing the model to learn more radiological features associated to each class. This is the typical modelling routine to maximize the data in model building when the dataset is limited, as in the case of COVID-19. In the second strategy, we pursued a more stringent model testing by splitting the dataset into 70% training, 20% validation, and 10% testing, congruent to the approach adopted in [4, 14, 15, 20, 21, 23]. The main difference between these strategies is the existence of the “unseen” dataset in the latter, i.e., data that were not used to train the model will be used as a test dataset mimicking the clinical deployment scenario. We note that our approach aims to illustrate quantitatively the effect of data distribution on the different techniques adopted in several works considering the limited number of COVID-19 cases. To our knowledge, no literature has designed a similar experiment in model development, i.e., previous studies utilized only either one of the two strategies.

To further evaluate the robustness and clinical applicability of the models, we introduce several variants of detection scenarios. These detection scenarios involve training the models to detect different class labels. A two-class detection scenario refers to the ability of the model to detect two classes: normal and pneumonia. A three-class detection refers to the ability of the model to detect three classes: normal, non-COVID-19 pneumonia, and COVID-19 pneumonia. Lastly, a four-class detection refers to the ability of the model to distinguish CXR images into one of the four classes: normal, bacterial pneumonia, viral pneumonia, and COVID-19 pneumonia. Following this design, a relabeling scheme guided by our resident radiologists were formulated and applied to our entire dataset. For the two-class detection scenario, class labels such as bacterial, viral, and COVID-19 were generalized as pneumonia. For the three-class detection scenario, labels such as bacterial and viral pneumonia from the external dataset and non-COVID-19 pneumonia from our internal dataset were reclassified and generalized as non-COVID-19 pneumonia. Tables 2 and 3 show the data summary for the different development strategy and detection scenarios.

**Table 2:** First strategy data distribution for different detection scenarios.

Two-class Detection			Three-class Detection			Four-class Detection		
Labels	Training	Validation	Labels	Training	Validation	Labels	Training	Validation
Normal	2,875	718	Normal	2,875	718	Normal	2,875	718
Pneumonia	4,333	1,083	Non-COVID-19 Pneumonia	3,837	959	Bacterial Pneumonia	2,229	557
-	-	-	COVID-19 Pneumonia	496	124	Viral Pneumonia	1,204	301
-	-	-	-	-	-	COVID-19 Pneumonia	496	124

**Table 3:** Second strategy data distribution for different detection scenarios.

Two-class Detection				Three-class Detection				Four-class Detection			
Labels	Training	Validation	Testing	Labels	Training	Validation	Testing	Labels	Training	Validation	Testing
Normal	2,514	719	360	Normal	2,514	719	360	Normal	2,514	719	360
Pneumonia	3,790	1,084	542	Non-COVID-19 Pneumonia	3,356	960	480	Bacterial Pneumonia	1,949	558	279
-	-	-	-	COVID-19 Pneumonia	434	124	62	Viral Pneumonia	1,053	301	151
-	-	-	-	-	-	-	-	COVID-19 Pneumonia	434	124	62

### Transfer learning

One of the main challenges in developing AI-driven disease detection models in the medical field is the scarcity of the dataset and the difficulty in acquisition due to data privacy and other ethical considerations. To circumvent the data limitation problem, the use of pre-trained models via transfer learning offers an alternative development workflow. Transfer learning is a machine learning technique in which models trained on a specific task is repurposed for a new task. Through the years, the development of deep learning models for disease detection via transfer learning has been widely adopted as a modelling strategy, particularly in cases where the dataset is limited. For instance, Narin et al. [20] used transfer learning to distinguish COVID-19 from normal images using only 100 CXR images. Albeit different base architecture selection and optimization methods, several studies [4, 14, 17, 18, 20, 23–25] have also adopted transfer learning in developing COVID-19 pneumonia detection models.

In our model development pipeline, five well-known architectures which were also adopted by several studies in COVID-19 detection were selected as the base architectures. These are InceptionV3 [4, 14, 15, 20, 21, 23, 24], Inception-ResNet V2 [17, 20, 21, 23–25], Xception [17, 18, 23], VGG19 and MobileNet [4, 14, 17, 23]. In training and fine-tuning the CNN architectures, we used stochastic gradient descent (SGD) as an optimizer and categorical cross-entropy as the loss function.

### Hyperparameter tuning and performance evaluation

Hyperparameter tuning plays a crucial part in optimizing the performance of the models. We implemented the grid search method to obtain the optimal values of the hyperparameters. Grid search is an exhaustive optimization procedure that involves permuting all the possible combinations of the selected hyperparameters to determine the values that would result to highest model performance. Table 4 shows the list of the selected hyperparameters and their corresponding values.

**Table 4:** Hyperparameters values used during model optimization.

Hyperparameter	Values
Batch Size	16, 32
Fully Connected Layers	256, 512, 1024
Dropout	0.2, 0.3, 0.5
Regularizer	L1(0.001, 0.01), L2(0.001, 0.01)

The general and per label performance of the models were evaluated using 10-fold cross validation to determine the best performing models considering the different data distribution and detection scenarios. General performance refers to the performance of the model considering the overall performance in all classes, while the per class performance refers to the performance of a model considering a particular class.

The detection models were developed using Tensorflow (TF) version 2.1.0 in Python 3.7 environment and optimized using a 32-core (64 Thread) computing server and two NVIDIA Tesla V100 (32GB) graphic processing units (GPU) servers. Depending on the complexity of the model and the selected hyperparameter combinations, e.g., InceptionV3 with batch size 32, fully connected layers of 256, dropout of 0.2, and regularizer of L2(0.001), the runtime ranges from 60 to 120 minutes per model permutation.

## Results

From a clinical point of view, COVID-19 pneumonia detection models should reliably identify positive cases and ensure that the predicted positive cases are true positive. This will allow healthcare frontliners to isolate positive cases during triaging and employ suitable mitigation strategies to effectively reduce the transmission rate. Moreover, the detection models should lessen the detection of false positives to reduce the resources allocated in testing negative cases. In this view, we considered a more realistic clinical deployment scenario and evaluated the performance of the models focusing on three key parameters: sensitivity (Sn), specificity (Sp), and positive predictive values (PPV). Sn and Sp refer to the ability of the model to detect positive and negative cases, respectively, while PPV is the probability that the subjects with positive screening results are true COVID-19 positive. While we also provide in the Supplementary section other metrics such as negative predictive value (NPV), F1 score, geometric mean (gmean) and likelihood ratio (±LR) for external evaluation of the models, we focus our analysis on the key parameters because in clinical deployment scenario, the COVID-19 detection model should be highly sensitive with high specificity and PPV.

### Development strategy

The development strategy we pursued aims to show the effect of the data distribution in optimizing the model. Driven by data limitation, earlier works opted to split the data into training and validation, a strategy that does not allow model testing on an unseen dataset. An alternative experimental design in building a detection model is to test the performance of the model on a dataset that is not included during the model training. Here we provide a quantitative comparison between these two approaches by calculating the standard deviation of the Sn, Sp, and PPV among the different models. Full numerical results are presented in the Supplementary section. The standard deviation of Sn, Sp, and PPV in all trained models in the first strategy are 4.61%, 1.31%, and 4.66%, respectively. For the second strategy, the standard deviation of Sn, Sp, and PPV are 4.98%, 1.29%, and 5.02%, respectively. These results highlight that despite the difference in data distribution between the two approaches, the general performances of the models are comparable, i.e., absolute difference in the standard deviation among the models in the two development strategies is at most 0.37%. At the current level of data available for modelling, the detection performance has less dependency on the quantity of data but more on the thorough hyperparameter search during model optimization. Table 5 shows the optimum hyperparameter values for the selected architectures along with the number of trainable parameters and average training runtime per epoch.

**Table 5:** Hyperparameters and the average optimization runtime per epoch of the best performing model.

Model	Batch Size	Fully Connected Layers	Regularizer	Dropout	Trainable Parameters	Runtime Per Epoch
InceptionV3	16	256	L2 (0.001)	0.2	23,015,331	$\tilde{5}6$ sec
InceptionResNetV2	32	512	L2 (0.001)	0.3	55,327,587	$\tilde{1}$ min 50 sec
Xception	16	256	L2 (0.001)	0.3	20,684,611	$\tilde{4}1$ sec
VGG	16	1024	L2 (0.001)	0.3	22,053,931	$\tilde{1}$ min 25 sec
MobileNet	16	1024	L2 (0.001)	0.5	3, 277,315	$\tilde{4}0$ sec

### Detection scenario

In our attempt to create a deployable model, the detection scenarios were designed to subdivide the multiclassification task to look for potential models that can be further evaluated to prove their applicability in a clinical setting. In this manner, we were able to evaluate the capabilities of the models in distinguishing different types of pneumonia from a normal CXR image. Our rigorous model training demonstrates that all optimized base architectures for two-class detection are highly capable of distinguishing pneumonia. The Sn, Sp, and PPV scores range from 93–96%, illustrating the high accuracy of the models to detect radiological features typically associated with pneumonia. Among the five models, InceptionV3 attained the highest performance in two-class classification with Sn, Sp, and PPV of 96%.

To specifically test the detection capability of the model in distinguishing COVID-19 from normal CXR and other types of pneumonia, we designed the three-class (normal, COVID-19, and non-COVID-19 pneumonia) and four class (normal, bacterial, viral, and COVID-19 pneumonia) detection scenarios. For the three-class detection scenario, the Sn, Sp, and PPV of the models range from 91–96%, 94–98%, and 90–96%, respectively. On the other hand, the Sn, Sp, and PPV for the four-class detection scenario range from 81–86%, 94–95%, and 81–86% indicating that the trained models performed better in three-class detection scenario. The significant reduction (~10%) in the sensitivity and PPV in differentiating COVID-19 pneumonia from bacterial and viral pneumonia in four-class detection scenario may indicate considerable similarities in radiological features among the different types of pneumonia. Interestingly, the performance scores illustrate that the Inception-based models generally outperformed MobileNet and VGG19. Furthermore, InceptionV3 attained the highest performance in detecting COVID-19 pneumonia with Sn, Sp, and PPV of 86%, 99%, and 91%, respectively. Table 6 shows the performance of trained models in COVID-19 pneumonia detection.

**Table 6:** Performance of various models in COVID-19 pneumonia detection.

Model	Sn (%)	Sp (%)	PPV (%)
InceptionV3	86	99	91
InceptionResNetV2	82	99	87
Xception	72	98	79
VGG	80	99	85
MobileNet	66	99	84

To further evaluate the detection capability of the InceptionV3 to distinguish COVID-19 pneumonia from other classes, the accuracy, F1-score, gmean, and confusion matrix were obtained. In terms of accuracy, the InceptionV3 has a generalization ability of 96% in identifying both the positive and negative cases. The F1-score and g-mean are both equal to 96%, indicating the balance between Sn and PPV as well as the classification performance on both majority and minority class, respectively. We note that similar works which also utilized InceptionV3 to develop a COVID-19 pneumonia detection model have achieved a sensitivity, PPV, accuracy, and F1-score of 91% [4], while [3] has achieved an accuracy of 93% to distinguished COVID-19 pneumonia, illustrating that our optimization procedure led to better performance. In addition, the per class performance in Table 7 shows that the InceptionV3 is highly sensitive and specific with high PPV on all classes.

**Table 7:** Per-class performance of the InceptionV3 model.

Class Labels	Sn (%)	Sp (%)	PPV (%)
Normal	97	97	96
Non-COVID-19 Pneumonia	99	97	99
COVID-19 Pneumonia	86	99	91

**Fig 2:**
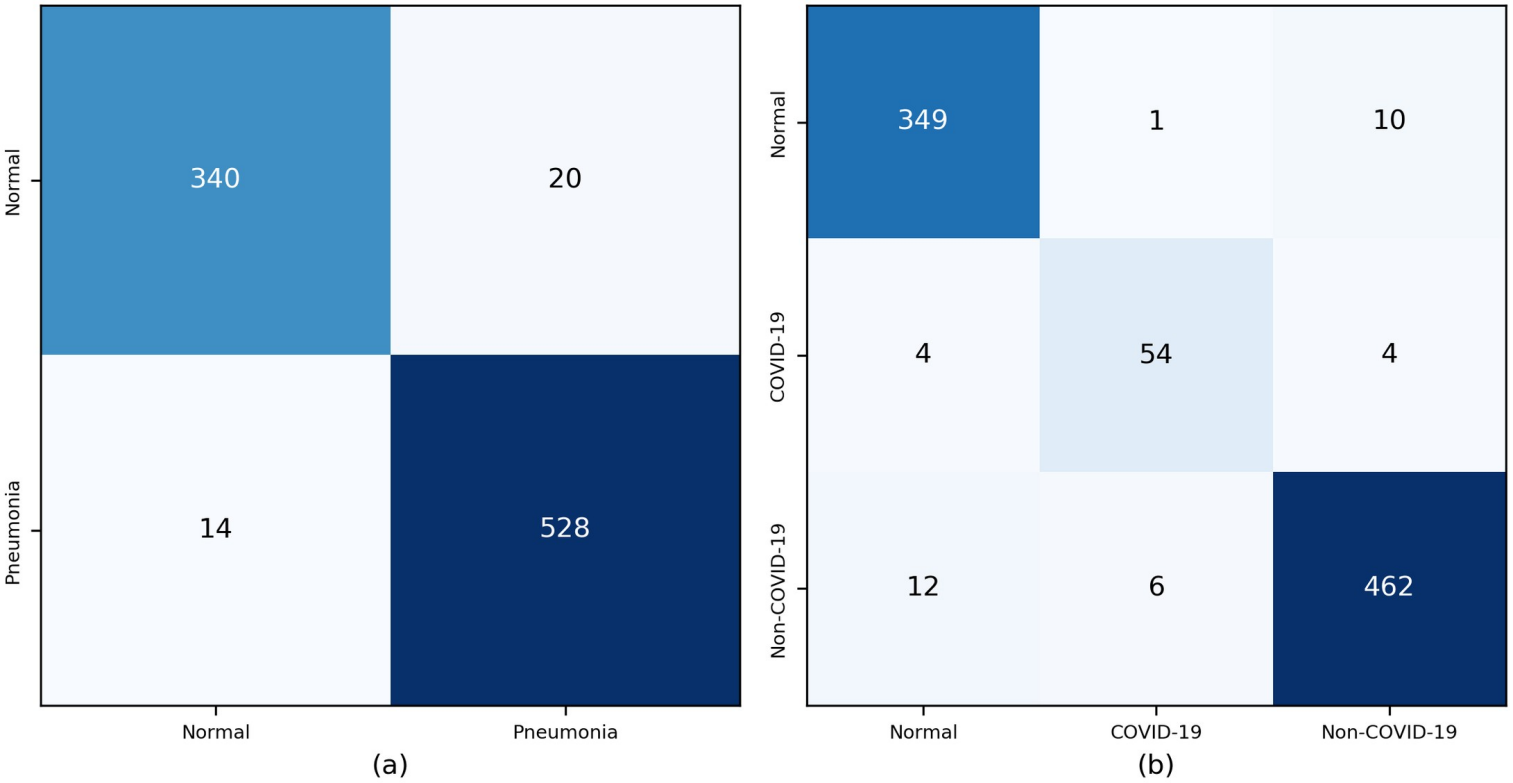
Confusion matrix of the InceptionV3 detection model. (a) two-class detection scenario and (b) three-class detection scenario.

**Fig 3:**
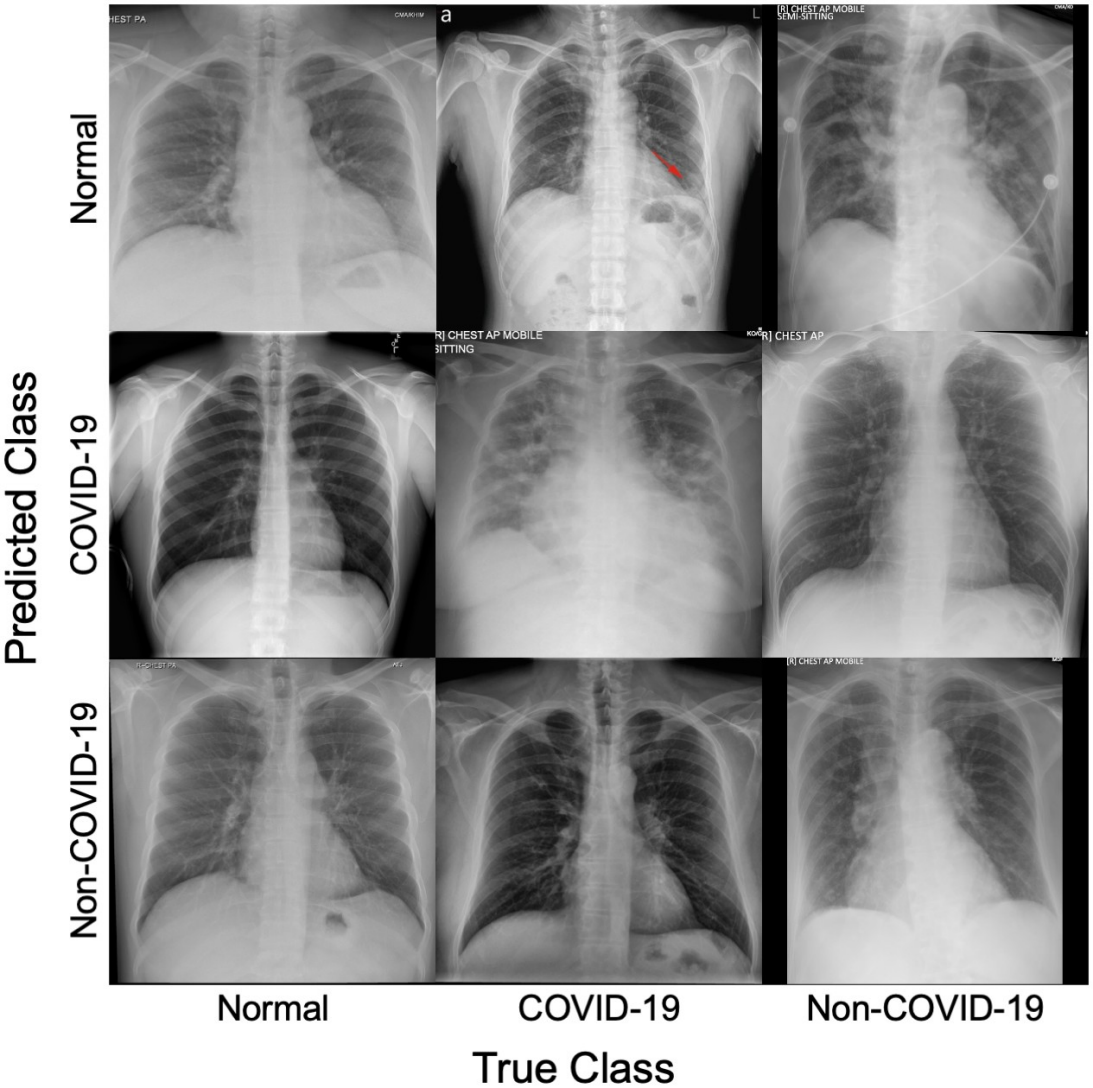
Example of test CXR images which were correctly classified and misclassified by the model.

Fig 2 shows the resulting confusion matrix for the two-class and the three-class detection scenarios. The IncetpionV3 was able to correctly predict 528 out of 548 (96%) unseen pneumonia CXR (Fig 2a) images for the two-class scenario. For the three-class detection scenario, the model was able to correctly predict 54 out of 61 (84%) unseen COVID-19 pneumonia positive cases. Furthermore, Fig 3 shows some of unseen dataset along with the true class and predicted class to demonstrate the capability of the model.

### Clinical deployment perspective

In general, to evaluate diagnostic the performance of AI-driven detection models designed to assist radiologists in analyzing CXR images, a multi-reader study is performed. Several comparative studies [26–28] have shown that the performance of AI-driven detection models is on par with practicing radiologist. However, we note that in clinical deployment scenario, the AI system should be adopted and integrated in clinical workflow as a decision support tool. This perspective has been illustrated in previous works [29, 30], in which the performance of the radiologists with and without the assistance of the AI system were compared. For example, Bai et al. [29] shows that the radiologists achieved better performance in differentiating COVID-19 pneumonia from other types of pneumonia with the assistance of AI. The probability scores provided by the AI improved the accuracy, sensitivity, and specificity: 85% to 90%, 79% to 88%, and 88% to 91%, respectively [29].

**Fig 4:**
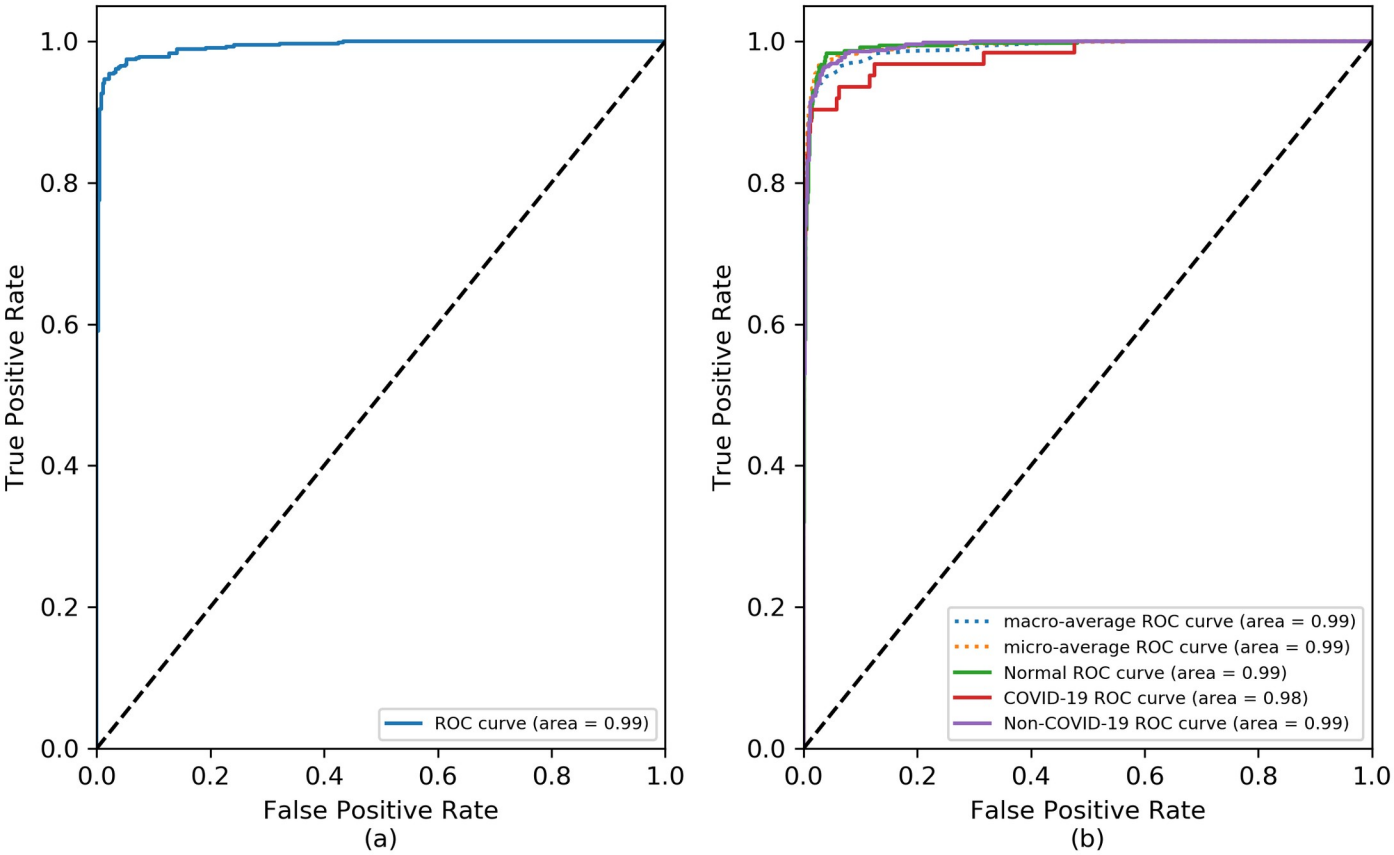
ROC curve of the top model. (a) pneumonia detection (two-class detection) and (b) COVID-19 detection (three-class detection).

Considering a prospective clinical validation study, we assess the diagnostic performance of our trained InceptionV3 as a screening tool by determining the receiver operating characteristics (ROC) and area under the curve (AUC). The ROC curves allow the visualization of the model’s ability to distinguish among classes at different thresholds whereas AUC measures the separability among the different classes. The higher the AUC, the better the model is at differentiating the COVID-19 pneumonia from other classes. More precisely, we evaluated the performance of the model in distinguishing pneumonia from normal CXR in two-class detection scenario as well as detecting COVID-19 pneumonia in three-class scenario. Compared with a similar work by Punn et. al. [9] which attained an AUC score of 0.90, our trained InceptionV3 detector achieved an AUC of 0.98 for COVID-19 class and a micro-average AUC of 0.99 for other classes. Moreover, our model achieved better performance with an AUC of 0.99 compared to 0.80 reported in the work [31] in which deep learning architectures and supervised classifiers were combined to develop model for pneumonia detection. Fig 4 shows the ROC curve of the top models for pneumonia detection and COVID-19 detection.

**Fig 5:**
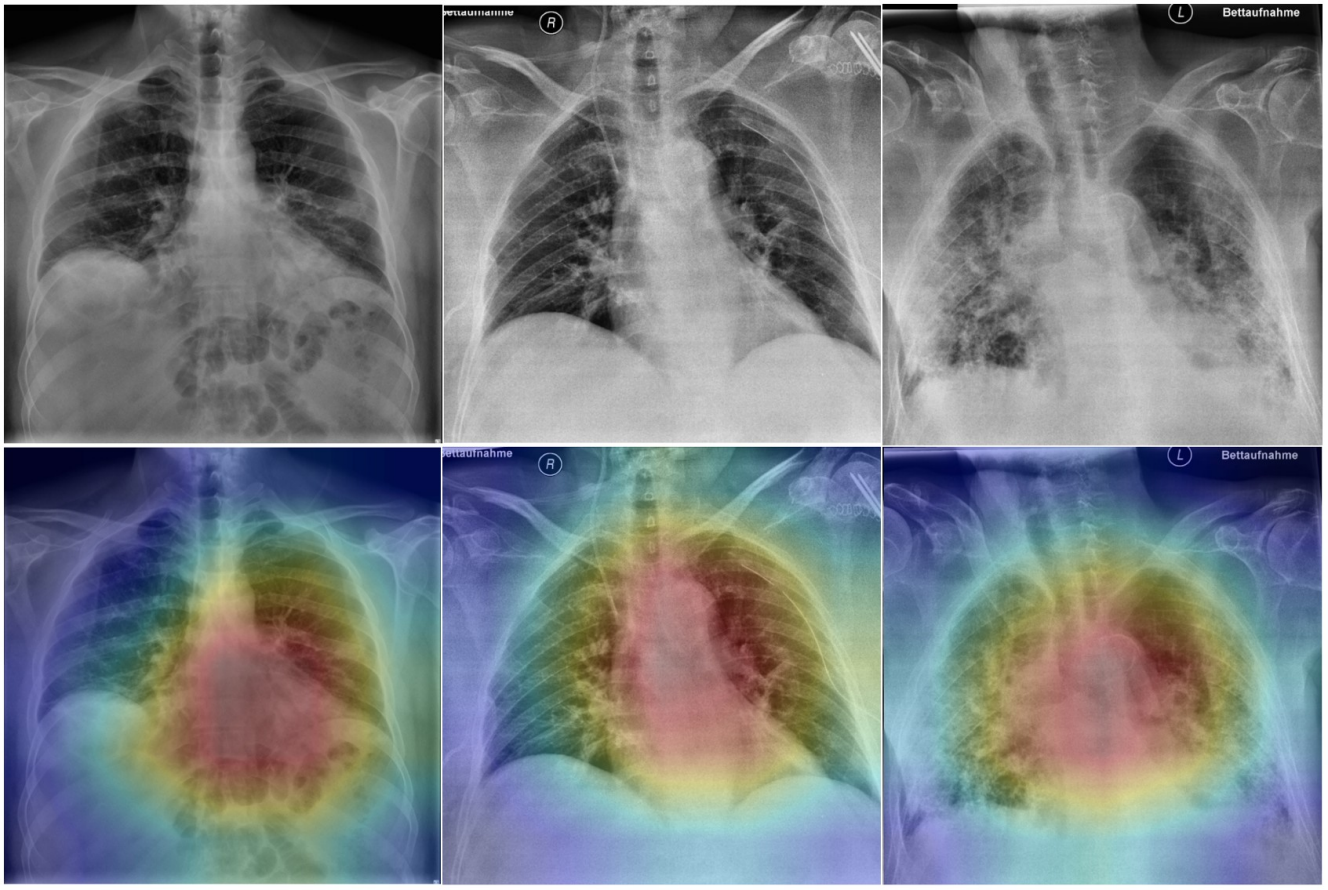
Grad-CAM for three CXR images diagnosed with COVID-19 pneumonia. The first column shows the original CXR, the second column shows the overlaid activation map on the original image.

The availability of a visual characterization of the model prediction provides the clinicians assistance in providing a final diagnosis. The explainability of AI-driven detection models is achieved using a heat map that illustrates the model’s decisions. The gradient information from the CXR images is fed back into the final convolutional layer to determine the importance of each neuron in classifying an image to each disease class [32]. Fig 5 shows the class activation maps of CXR images from COVID-19 cases.

One of the technical challenges in AI deployment is the model’s compatibility from the development environment to an actual clinical translation. The wide variety of deployment tools and dependencies often results in restructuring of the model which may lead to inconsistencies during model translation. Another technical issue is the lack of model portability in which difficulty arises when migrating AI models to another host, e.g. local machine to cloud environment. In this perspective, we evaluated the runtime of the model when deployed to different computational infrastructure such as computer desktop, local server, or cloud environment. See Table 8 in S1 Appendix for the hardware specification. The prediction runtime in which the AI model analyzes an image and subsequently provides the corresponding prediction score is between 15 seconds to 2 minutes. Evidently, deployment of our AI-driven model to facilitate screening of COVID-19 pneumonia is attainable using a typical computer desktop and therefore deemed scalable even in remote hospitals where computational infrastructure and cloud services are inaccessible.

## Conclusion

In this study, we developed AI-driven models designed for COVID-19 pneumonia detection using CXR images. To augment the existing CXR dataset available in open access repositories, we conducted a retrospective clinical study in which 1,171 clinically validated CXR images across 821 cohorts were generated. In optimizing the detection models, we pursued two different strategies to assess the impact of the data distribution and provide a quantitative comparison between two study designs adopted in previous works. In our attempt to build robust and deployable model for clinical use, we introduce different detection scenarios. The diagnostic performance of the detection models was evaluated considering key parameters relevant to clinical deployment standpoint. At the current level of data available for modelling, we have developed highly sensitive and specific with high PPV models that can easily classify pneumonia from normal CXR as well as distinguish COVID-19 pneumonia from other types of pneumonia. Furthermore, we illustrated that our AI-driven detection model can be deployed in a typical computer desktop with an approximate runtime of two minutes to analyze an image; hence, deemed scalable and can facilitate automated screening of COVID-19 cases in remote areas. To a large extent, this study provides clinically validated CXR images and a well-formulated study design which can be adopted by to the research community to advance and create practical AI solutions to mitigate COVID-19. Naturally, the research design can be extended to other types of pneumonia and pulmonary diseases depending on the available dataset and clinical context.

## Supporting information

S1 Appendix (DOCX)
