# Supplementary material for: Artificial intelligence on COVID-19 pneumonia detection using chest xray images
Source: PLoS One. 2021 Oct 14;16(10):e0257884. doi: 10.1371/journal.pone.0257884 (PMC8516252; doi:10.1371/journal.pone.0257884)
Supplement: S1 Appendix — (DOCX) [file pone.0257884.s001.docx]

**Table 1. Summary statistics of the metadata of the internally generated dataset**

| **Feature** | **Description** |
| --- | --- |
| Age | 23-100 |
| Sex | Male: 430, Female: 391 |
| Weight (kg) | 39kg - 118kg |
| Height (m) | 1.0 m - 1.98 m |
| BMI | 17.94 - 61.2 |
| Systolic Blood Pressure (mmHg) | 10 - 253 |
| Diastolic Blood Pressure (mmHg) | 40 - 150 |
| Heart Rate (beats per minute) | 25 - 170 |
| Respiratory Rate (breath per minute) | 14-145 |
| Temperature (C) | 32 - 41.2 |
| O2 Saturation (%) | 77-100 |
| P/F Ratio | 60-664.1 |

**Table 2. Summary of External Data Sources**

| **Source** | **Label** | **No. Images** |
| --- | --- | --- |
| Shenzhen Hospital Dataset | Normal Tuberculosis | 662 |
| Montgomery County Dataset | Normal Tuberculosis | 138 |
| India Dataset | Normal Tuberculosis | 155 |
| Chest X-Ray Images (Pneumonia) Dataset | Normal Bacterial Pneumonia Viral Pneumonia | 5, 877 |
| COVID Chest X-Ray Dataset | COVID | 444 |
| COVID-19 Radiography Dataset | COVID | 15 |
| Figure-1 COVID Chest X-ray Dataset | COVID | 93 |

**Table 3.1. General performance of the first strategy for all detection scenarios using InceptionV3 as base model**

| **Metric** | **TRAINING** | | | **VALIDATION** | | |
| --- | --- | --- | --- | --- | --- | --- |
|  | 2-class | 3-class | 4-class | 2-class | 3-class | 4-class |
| Sensitivity | 96 | 95 | 88 | 95 | 94 | 86 |
| PPV | 96 | 95 | 88 | 95 | 94 | 86 |
| G-mean | 96 | 95 | 92 | 95 | 95 | 90 |
| F1 Score | 96 | 95 | 88 | 95 | 94 | 86 |
| Accuracy | 96 | 95 | 88 | 95 | 94 | 86 |

**Table 3.2. General performance of the second strategy for all detection scenarios using InceptionV3 as base model**

| **Metric** | **TRAINING** | | | **VALIDATION** | | | **TESTING** | | |
| --- | --- | --- | --- | --- | --- | --- | --- | --- | --- |
|  | 2-class | 3-class | 4-class | 2-class | 3-class | 4-class | 2-class | 3-class | 4-class |
| Sensitivity | 97 | 96 | 89 | 96 | 95 | 85 | 96 | 96 | 86 |
| PPV | 97 | 96 | 89 | 96 | 95 | 85 | 96 | 96 | 86 |
| G-mean | 97 | 96 | 91 | 95 | 95 | 88 | 96 | 96 | 89 |
| F1 Score | 97 | 96 | 89 | 96 | 95 | 85 | 96 | 96 | 86 |
| Accuracy | 97 | 96 | 89 | 96 | 95 | 85 | 96 | 96 | 86 |

**Table 3.3. Per class performance of different detection scenarios using InceptionV3 as base model**

| **2-class** | | | | | | | **3-class** | | | | | | | **4-class** | | | | | | |
| --- | --- | --- | --- | --- | --- | --- | --- | --- | --- | --- | --- | --- | --- | --- | --- | --- | --- | --- | --- | --- |
| **Labels** | **Sn** | **Sp** | **PPV** | **NPV** | **LR+** | **LR-** | **Labels** | **Sn** | **Sp** | **PPV** | **NPV** | **LR+** | **LR-** | **Labels** | **Sn** | **Sp** | **PPV** | **NPV** | **LR+** | **LR-** |
| Normal | 94 | 97 | 96 | 96 | 36.56 | 0.06 | Normal | 97 | 97 | 96 | 98 | 32.84 | 0.03 | Normal | 96 | 96 | 95 | 97 | 23.71 | 0.04 |
| Pneumonia | 97 | 94 | 96 | 96 | 17.54 | 0.03 | Non-COVID-19 | 99 | 97 | 99 | 96 | 29.01 | 0.04 | Bacterial | 89 | 90 | 82 | 94 | 9.06 | 0.13 |
| - | - | - | - | - | - | - | COVID-19 | 86 | 99 | 91 | 99 | 104.52 | 0.13 | Viral | 63 | 95 | 71 | 91 | 11.35 | 0.44 |
| - | - | - | - | - | - | - | - | - | - | - | - | - | - | COVID-19 | 85 | 99 | 88 | 99 | 92.83 | 0.18 |

**Table 4.1. General performance of the first strategy for all detection scenarios using InceptionResNetV2 as base model**

| **Metric** | **TRAINING** | | | **VALIDATION** | | |
| --- | --- | --- | --- | --- | --- | --- |
|  | 2-class | 3-class | 4-class | 2-class | 3-class | 4-class |
| Sensitivity | 97 | 95 | 88 | 95 | 93 | 85 |
| PPV | 97 | 95 | 87 | 95 | 92 | 85 |
| G-mean | 96 | 95 | 91 | 94 | 93 | 89 |
| F1 Score | 97 | 95 | 87 | 95 | 94 | 85 |
| Accuracy | 97 | 95 | 88 | 95 | 93 | 85 |

**Table 4.2. General performance of the second strategy for all detection scenarios using InceptionResNeV2 as base model**

| **Metric** | **TRAINING** | | | **Validation** | | | **Testing** | | |
| --- | --- | --- | --- | --- | --- | --- | --- | --- | --- |
|  | 2-class | 3-class | 4-class | 2-class | 3-class | 4-class | 2-class | 3-class | 4-class |
| Sensitivity | 96 | 95 | 87 | 95 | 93 | 85 | 95 | 94 | 85 |
| PPV | 96 | 95 | 87 | 95 | 93 | 85 | 95 | 94 | 85 |
| G-mean | 96 | 96 | 91 | 95 | 94 | 89 | 94 | 95 | 89 |
| F1 Score | 96 | 95 | 87 | 95 | 93 | 85 | 95 | 94 | 85 |
| Accuracy | 96 | 95 | 87 | 95 | 93 | 85 | 95 | 94 | 85 |

**Table 4.3. Per class performance of different detection scenarios using InceptionResNetV2 as base model**

| **2-class** | | | | | | | **3-class** | | | | | | | **4-class** | | | | | | |
| --- | --- | --- | --- | --- | --- | --- | --- | --- | --- | --- | --- | --- | --- | --- | --- | --- | --- | --- | --- | --- |
| **Labels** | **Sn** | **Sp** | **PPV** | **NPV** | **LR+** | **LR-** | **Labels** | **Sn** | **Sp** | **PPV** | **NPV** | **LR+** | **LR-** | **Labels** | **Sn** | **Sp** | **PPV** | **NPV** | **LR+** | **LR-** |
| Normal | 91 | 97 | 96 | 94 | 35.38 | 0.09 | Normal | 94 | 96 | 95 | 96 | 26.7 | 0.07 | Normal | 97 | 95 | 94 | 97 | 23.71 | 0.04 |
| Pneumonia | 97 | 91 | 94 | 96 | 11.31 | 0.03 | Non-COVID-19 | 96 | 95 | 95 | 96 | 18.5 | 0.04 | Bacterial | 86 | 90 | 81 | 94 | 9.06 | 0.13 |
| - | - | - | - | - | - | - | COVID-19 | 82 | 99 | 91 | 98 | 45.48 | 0.25 | Viral | 60 | 94 | 70 | 91 | 11.35 | 0.44 |
| - | - | - | - | - | - | - | - | - | - | - | - | - | - | COVID-19 | 79 | 99 | 85 | 99 | 92.83 | 0.18 |

**Table 5.1. General performance of the first strategy for all detection scenarios using Xception as base model**

| **Metric** | **TRAINING** | | | **VALIDATION** | | |
| --- | --- | --- | --- | --- | --- | --- |
|  | 2-class | 3-class | 4-class | 2-class | 3-class | 4-class |
| Sensitivity | 94 | 92 | 83 | 92 | 92 | 84 |
| PPV | 94 | 92 | 83 | 92 | 92 | 83 |
| G-mean | 94 | 93 | 88 | 92 | 93 | 88 |
| F1 Score | 94 | 92 | 83 | 92 | 92 | 83 |
| Accuracy | 94 | 92 | 83 | 92 | 92 | 84 |

**Table 5.2. General performance of the second strategy for all detection scenarios using Xception as base model**

| **Metric** | **TRAINING** | | | **Validation** | | | **Testing** | | |
| --- | --- | --- | --- | --- | --- | --- | --- | --- | --- |
|  | 2-class | 3-class | 4-class | 2-class | 3-class | 4-class | 2-class | 3-class | 4-class |
| Sensitivity | 93 | 93 | 81 | 92 | 93 | 81 | 93 | 93 | 81 |
| PPV | 93 | 92 | 81 | 92 | 92 | 81 | 93 | 93 | 81 |
| G-mean | 93 | 94 | 87 | 92 | 93 | 86 | 93 | 94 | 86 |
| F1 Score | 93 | 92 | 81 | 92 | 92 | 81 | 93 | 93 | 81 |
| Accuracy | 93 | 93 | 81 | 92 | 93 | 81 | 93 | 93 | 81 |

**Table 5.3. Per class performance of different detection scenarios using Xception as base model**

| **2-class** | | | | | | | **3-class** | | | | | | | **4-class** | | | | | | |
| --- | --- | --- | --- | --- | --- | --- | --- | --- | --- | --- | --- | --- | --- | --- | --- | --- | --- | --- | --- | --- |
| **Labels** | **Sn** | **Sp** | **PPV** | **NPV** | **LR+** | **LR-** | **Labels** | **Sn** | **Sp** | **PPV** | **NPV** | **LR+** | **LR-** | **Labels** | **Sn** | **Sp** | **PPV** | **NPV** | **LR+** | **LR-** |
| Normal | 92 | 94 | 91 | 95 | 14.57 | 0.09 | Normal | 94 | 94 | 91 | 96 | 16.04 | 0.06 | Normal | 92 | 93 | 91 | 94 | 14.46 | 0.09 |
| Pneumonia | 94 | 92 | 95 | 91 | 10.88 | 0.07 | Non-COVID-19 | 94 | 95 | 96 | 93 | 38.10 | 0.28 | Bacterial | 81 | 91 | 82 | 90 | 29.94 | 0.25 |
| - | - | - | - | - | - | - | COVID-19 | 72 | 98 | 79 | 98 | 21.98 | 0.07 | Viral | 62 | 92 | 63 | 92 | 6.60 | 0.44 |
| - | - | - | - | - | - | - | - | - | - | - | - | - | - | COVID-19 | 74 | 98 | 71 | 98 | 9.08 | 0.23 |

**Table 6.1. General performance of the first strategy for all detection scenarios using MobileNet as base model**

| **Metric** | **TRAINING** | | | **VALIDATION** | | |
| --- | --- | --- | --- | --- | --- | --- |
|  | 2-class | 3-class | 4-class | 2-class | 3-class | 4-class |
| Sensitivity | 94 | 92 | 83 | 93 | 91 | 83 |
| PPV | 94 | 92 | 83 | 93 | 91 | 83 |
| G-mean | 94 | 93 | 88 | 92 | 92 | 88 |
| F1 Score | 94 | 92 | 83 | 93 | 91 | 83 |
| Accuracy | 94 | 92 | 83 | 93 | 91 | 83 |

**Table 6.2. General performance of the second strategy for all detection scenarios using MobileNet as base model**

| **Metric** | **TRAINING** | | | **Validation** | | | **Testing** | | |
| --- | --- | --- | --- | --- | --- | --- | --- | --- | --- |
|  | 2-class | 3-class | 4-class | 2-class | 3-class | 4-class | 2-class | 3-class | 4-class |
| Sensitivity | 94 | 91 | 84 | 93 | 91 | 83 | 93 | 91 | 84 |
| PPV | 94 | 91 | 84 | 93 | 90 | 82 | 93 | 90 | 83 |
| G-mean | 94 | 93 | 88 | 92 | 92 | 87 | 93 | 92 | 88 |
| F1 Score | 94 | 91 | 83 | 93 | 90 | 82 | 93 | 90 | 83 |
| Accuracy | 94 | 91 | 84 | 93 | 91 | 83 | 93 | 91 | 84 |

**Table 6.3. Per class performance of different detection scenarios using MobileNet as base model**

| **2-class** | | | | | | | **3-class** | | | | | | | **4-class** | | | | | | |
| --- | --- | --- | --- | --- | --- | --- | --- | --- | --- | --- | --- | --- | --- | --- | --- | --- | --- | --- | --- | --- |
| **Labels** | **Sn** | **Sp** | **PPV** | **NPV** | **LR+** | **LR-** | **Labels** | **Sn** | **Sp** | **PPV** | **NPV** | **LR+** | **LR-** | **Labels** | **Sn** | **Sp** | **PPV** | **NPV** | **LR+** | **LR-** |
| Normal | 93 | 93 | 90 | 95 | 14.01 | 0.07 | Normal | 92 | 93 | 90 | 95 | 13.88 | 0.08 | Normal | 95 | 93 | 91 | 96 | 13.39 | 0.05 |
| Pneumonia | 93 | 93 | 95 | 90 | 13.44 | 0.07 | Non-COVID-19 | 95 | 92 | 63 | 94 | 11.74 | 0.06 | Bacterial | 85 | 91 | 82 | 92 | 9.15 | 0.17 |
| - | - | - | - | - | - | - | COVID-19 | 66 | 99 | 84 | 97 | 30.97 | 0.49 | Viral | 61 | 94 | 66 | 92 | 9.71 | 0.42 |
| - | - | - | - | - | - | - | - | - | - | - | - | - | - | COVID-19 | 67 | 99 | 86 | 98 | 76.45 | 0.33 |

**Table 7.1. General performance of the first strategy for all detection scenarios using VGG as base model**

| **Metric** | **TRAINING** | | | **VALIDATION** | | |
| --- | --- | --- | --- | --- | --- | --- |
|  | 2-class | 3-class | 4-class | 2-class | 3-class | 4-class |
| Sensitivity | 96 | 95 | 87 | 95 | 94 | 85 |
| PPV | 96 | 95 | 87 | 95 | 93 | 85 |
| G-mean | 96 | 96 | 91 | 94 | 94 | 89 |
| F1 Score | 96 | 95 | 87 | 95 | 93 | 85 |
| Accuracy | 96 | 95 | 87 | 95 | 94 | 85 |

**Table 7.2. General performance of the second strategy for all detection scenarios using VGG as base model**

| **Metric** | **TRAINING** | | | **Validation** | | | **Testing** | | |
| --- | --- | --- | --- | --- | --- | --- | --- | --- | --- |
|  | 2-class | 3-class | 4-class | 2-class | 3-class | 4-class | 2-class | 3-class | 4-class |
| Sensitivity | 96 | 95 | 87 | 95 | 93 | 84 | 95 | 93 | 85 |
| PPV | 96 | 95 | 87 | 95 | 93 | 84 | 95 | 94 | 85 |
| G-mean | 96 | 96 | 91 | 95 | 94 | 89 | 95 | 95 | 89 |
| F1 Score | 96 | 95 | 87 | 95 | 93 | 84 | 95 | 93 | 85 |
| Accuracy | 96 | 95 | 87 | 95 | 93 | 84 | 95 | 93 | 85 |

**Table 7.3. Per class performance of different detection scenarios using VGG as base model**

| **2-class** | | | | | | | **3-class** | | | | | | | **4-class** | | | | | | |
| --- | --- | --- | --- | --- | --- | --- | --- | --- | --- | --- | --- | --- | --- | --- | --- | --- | --- | --- | --- | --- |
| **Labels** | **Sn** | **Sp** | **PPV** | **NPV** | **LR+** | **LR-** | **Labels** | **Sn** | **Sp** | **PPV** | **NPV** | **LR+** | **LR-** | **Labels** | **Sn** | **Sp** | **PPV** | **NPV** | **LR+** | **LR-** |
| Normal | 93 | 96 | 94 | 96 | 25.29 | 0.07 | Normal | 94 | 96 | 95 | 96 | 26.86 | 0.06 | Normal | 93 | 96 | 94 | 95 | 21.87 | 0.07 |
| Pneumonia | 96 | 93 | 96 | 94 | 14.45 | 0.04 | Non-COVID-19 | 92 | 95 | 98 | 93 | 24.62 | 0.07 | Bacterial | 85 | 93 | 85 | 92 | 11.34 | 0.18 |
| - | - | - | - | - | - | - | COVID-19 | 80 | 99 | 85 | 99 | 26.59 | 0.15 | Viral | 65 | 92 | 64 | 92 | 8.12 | 0.38 |
| - | - | - | - | - | - | - | - | - | - | - | - | - | - | COVID-19 | 87 | 98 | 81 | 99 | 52.93 | 0.13 |

Table 8. Estimated prediction runtime for different computational infrastructure setup.

| **Machine** | **Specifications** | **Run time** |
| --- | --- | --- |
| AWS | 2 virtual central processing units (vCPUs) and 8GB memory | ~ 1 min |
| Typical Desktop Machine | 2.3GHz Intel Core i5 and 8GB memory | 1-2 min |
| Local Server | 32-core (64 Thread) computing server and two 181  NVIDIA Tesla V100 (32GB) graphic processing units (GPU) servers. | ~15 sec |


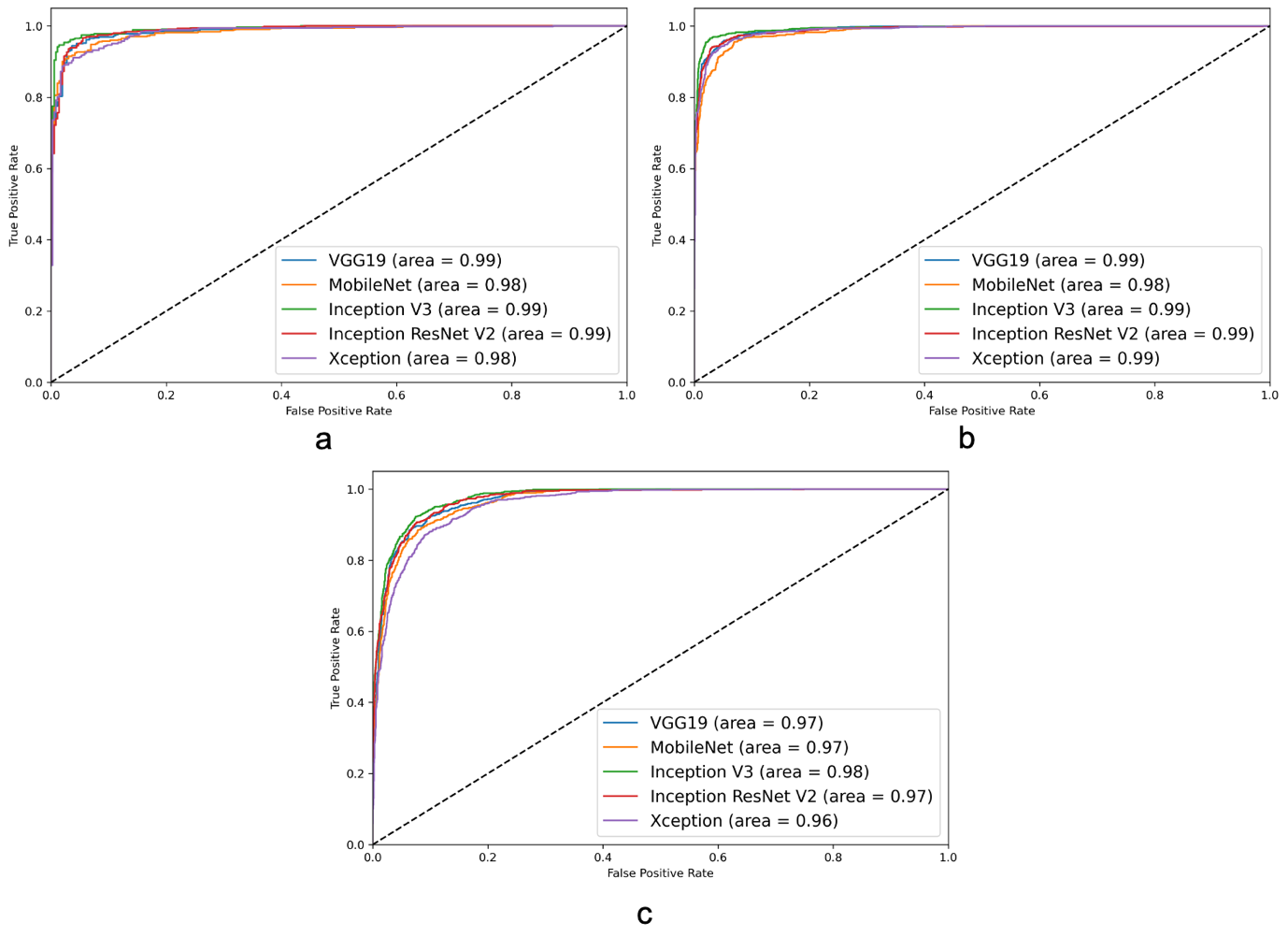


**Figure 1. ROC curves of the optimized models for (a) 2-class, (b) 3-class, (c) 4-class**
